# Epigenetic and sex differences in opioid use disorder in chronic pain: A real‐world study linked with *OPRM1* DNA methylation

**DOI:** 10.1111/adb.13422

**Published:** 2024-07-01

**Authors:** Laura Agulló, Mónica Escorial, Samantha Orutño, Javier Muriel, Juan Sandoval, César Margarit, Ana M. Peiró

**Affiliations:** ^1^ Pharmacogenetic Unit, Clinical Pharmacology Department Alicante Institute for Health and Biomedical Research (ISABIAL) Alicante Spain; ^2^ Bioengineering Institute, Department of Pharmacology, Paediatrics and Organic Chemistry Miguel Hernández University (UMH) Elche Spain; ^3^ Alicante Institute for Health and Biomedical Research (ISABIAL) Alicante Spain; ^4^ Epigenomics Unit La Fe Health Research Institute Valencia Spain; ^5^ Pain Unit, Department of Health of Alicante Dr. Balmis General Hospital Alicante Spain

**Keywords:** chronic pain, DNA methylation, epigenetics, opioid use disorder, *OPRM1* gene, pharmacogenetics, sex differences

## Abstract

Opioid use disorder (OUD) is a multifaceted condition influenced by sex, genetic and environmental factors that could be linked with epigenetic changes. Understanding how these factors interact is crucial to understand and address the development and progression of this disorder. Our aim was to elucidate different potential epigenetic and genetic mechanisms between women and men that correlate with OUD under real‐world pain unit conditions. Associations between analgesic response and the DNA methylation level of the *opioid mu receptor* (*OPRM1*) gene (CpG sites 1–5 selected in the promoter region) were evaluated in 345 long opioid‐treated chronic non cancer pain: cases with OUD (*n* = 67) and controls (without OUD, *n* = 278). Cases showed younger ages, low employment status and quality of life, but higher morphine equivalent daily dose and psychotropic use, compared to the controls. The patients with OUD showed a significant decrease in *OPRM1* DNA methylation, which correlated with clinical outcomes like pain relief, depression and different adverse events. Significant differences were found at the five CpG sites studied for men, and exclusively in women for CpG site 3, in relation to OUD diagnosis. These findings support the importance of epigenetics and sex as biological variables to be considered toward efficient OUD understanding and therapy development.

## Graphical abstract

Cases with opioid use disorder (OUD) were younger ages, low employment status and quality of life, but higher morphine equivalent daily dose and psychotropic use. The patients with OUD showed a significant decrease in *OPRM1* DNA methylation, which correlated with clinical outcomes like pain relief, depression and different adverse events. Sex‐differences were found at the CpG sites studied related to OUD.

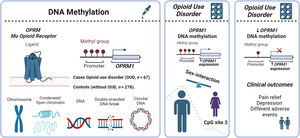

## 1 INTRODUCTION

Opioid use disorder (OUD) affects millions of chronic non cancer pain (CNCP) people worldwide^1^ and requires the evaluation of different biological variables, including sex.^2^ Findings from the literature suggest that women are more likely to use prescription opioids^3^ with faster progression of abuse after their first substance use, unlike men who show higher overdose death rates.^4^ Taking into account the higher chronic pain prevalence in older women,^5^ the socio‐cultural influence in pain response,^6^ and the existing gender inequalities in pain management,^7^ understanding sex differences seems essential for effective OUD prevention.^8^, ^9^

Some genetic markers and epigenetic modifications have been widely studied for their association in a variety of drug addiction^10^, ^11^ and pain sensitivity phenotypes,^12^ most notably μ*‐opioid receptor 1* (*OPRM1*, A118G, rs1799971‐G allele).^13^ The persistence of long‐term opioid use neuroadaptations can be mediated partly by the epigenetic remodelling of gene expression programs. In fact specific epigenetic modifications, such as DNA methylation patterns at a few CpG sites^14^, ^15^ have been associated with addictive behavioural and modulation of reward circuitry. Previous data from our laboratory suggest that an increase in *OPRM1* gene methylation is linked with a milder impact of the G‐variant allele in morphine equivalent daily dose (MEDD) reduction, an observation that is stronger in men versus women.^16^ In addition, such an increase could be associated with the impact on compulsive opioid behaviours and the OUD risk.^17^, ^18^ Hence, the possible involvement of a sex‐mediated genetic–epigenetic interaction could be considered to be a modulator factor.^19^, ^20^, ^21^ This may link environmental stimuli and genetic impact.^22^, ^23^ However, the extent to which these adaptations differ between women and men, along with the underlying mechanisms, remains unclear.^24^

The aim of this study was to explore sex‐related differences linked with the DNA methylation/genotypes that may affect *OPRM1* gene expression to, thus, condition differential OUD risks between sexes.

## 2 MATERIAL AND METHODS

### 2.1 Study design

An observational cross‐sectional study was designed and conducted at the Pain Unit (PU) in the Alicante Health Department at the Dr. Balmis General University Hospital (Spain) from October 2021 to September 2022. This study was approved by the Ethics Committee Board of the Dr. Balmis General University Hospital of Alicante (code: PI2023/018). Subjects gave verbal and signed informed consent before participating in interviews. The confidentiality of all the information was guaranteed. The study conforms to the Declaration of Helsinki regarding research involving human subjects. The generated datasets are available from the corresponding author upon reasonable request.

### 2.2 Participants and data collection

Present study analysed samples from 345 long‐term, opioid‐treated chronic non‐cancer pain patients (OUD cases, *n* = 67; or CNCP controls, *n* = 278) obtained from Biobank (Alicante Institute for Health and Biomedical Research [ISABIAL], Spain). This study adhered to the Spanish National Biobanks Network. Data were collected from the original databases and completed from patients' electronic health records (EHRs). The inclusion criteria were patients aged ≥18 years old and suffering chronic non cancer musculoskeletal pain (moderate or severe pain lasting at least 6 months) with long‐term opioids (≥3–6 months). Exclusion criteria were cancer pain, neuropathic pain, such as trigeminal neuralgia, mononeuritis and multiple sclerosis^25^, ^26^ or mixed pain (e.g., migraine, headache, cervicalgia, nontraumatic compartment syndrome), or were an opioid prescription for <6 months.^25^, ^26^

#### 2.2.1 Clinical and hospital resources use outcomes

A Global Pain State questionnaire measuring qualitatively pain intensity, relief and quality of life was collected at the time of interviews. Pain intensity and relief were measured using the Visual Analogue Scale (VAS), which consists of a horizontal line ranging from 0 (lowest) to 100 mm (highest), where the patient points on the line according to the pain or relief intensity that (s)he feels, respectively. Other demographic characteristics, such as age, sex, and employment status (works, retired, work disability, unemployed or homemaker), were also recorded.

Quality of life was evaluated with the EuroQol‐5D‐3L scale, which consists of a VAS from 0 (the worst imaginable health status) to 100 mm (the best imaginable), where patients indicate their actual health utility status (0 death to 1 perfect health) using mobility, self‐care, usual activities, pain/discomfort and anxiety/depression dimensions (permission code 53112 https://euroqol.org/). Psychological status was calculated by the Hospital Anxiety and Depression Scale (HADS). It consists of 0–21 scores that are classified as normal (<7), probable (8–10) and case (>11 scores).

Moreover, the use (yes/no) of simple analgesics (i.e., paracetamol and metamizole), nonsteroidal anti‐inflammatory drugs (NSAIDs) and opioids (i.e., tramadol, codeine, fentanyl, oxycodone, tapentadol, buprenorphine, morphine, hydromorphone and methadone), along with immediate release opioids, was recorded. In different combinations of opioids, MEDD was estimated using available references. The number of adverse events (AEs) was collected with a list of the most frequent analgesic side effects from the Summary of Product Characteristics frequency as ‘very common’ or ‘common’, together with a blank field to add any other developed AE/AEs. Other parameters were Hospital Frequentation, which included hospital admission, emergency department visits and drug change prescription.

### 2.3 Epigenetic and genetic data analysis

At the time of enrolment, the patient samples were collected for the pharmacogenetic analysis. Approximately 2 mL of saliva was collected in tubes containing 5 mL of PBS 1× and stored at −80°C until processing. Genomic DNA was isolated using the E.N.Z.A. forensic DNA kit (Omega Bio‐Tek Inc., USA) in accordance with the manufacturer's instructions.

#### 2.3.1 Pharmacogenetic analysis

Gene variants were genotyped for the *OPRM1* (rs1799971) variants following the real‐time PCR rotor gene Q system (Qiagen, Germany) with the use of specific TaqMan MGB® probes (Applied Biosystems, USA) (Table S1). The amplification parameters were as follows: denaturation step of 10 min at 95°C, 40 cycles for 15 s denaturation at 92°C and a 1‐min final extension at 60°C.

#### 2.3.2 DNA methylation analysis

First, a DNA integrity quality analysis was performed to ensure that DNA met the required standard quality criteria. All the DNA samples were quantified by the fluorometric method (Quan‐iT PicoGreen DsDNA Assay, Life Technologies) and assessed for purity using a NanoDrop 2000c (Thermo Fisher Scientific) with the 260/280 and 260/230 ratio measurements. The obtained high‐quality DNA samples (500 ng) were selected for bisulfite conversion using the EZ DNA Methylation kit (Zymo Research Corp.) following the manufacturer's recommendations.

A triplet of primers was designed for each promoter region of the *OPRM1* gene using the Qiagen's PyroMark Assay Design 2.0 software to hybridize to the CpG‐free sites to ensure methylation‐independent amplification and sequencing steps. The primer sequences are listed in Table 1.

**TABLE 1:** Demographic and clinical analysis by sex.

	Women (*n* = 194)		Men (*n* = 151)	
	Controls 163 (84%)	Cases OUD 31 (16%)	Controls 115 (76%)	Cases OUD 36 (24%)
Age (years old)	**67 [57–76]** **, ^**+**^	**60 [46–69]** ^**+**^	**60 [52–74]** **	50 [44–57]
Employment status (%)				
Working	12	11	13	4
Retired	**52** *	18	**54** *	32
Work disability	18	**39** *	27	**56** *
Unemployed	4	11	6	4
Clinical outcomes				
Pain intensity (VAS, 0–100 mm)	60	63	60	63
Pain relief (VAS, 0–100 mm)	33	43	27	39
Quality of life (VAS, 0–100 mm)	46	41	**49** **	36
HAD depression (0–21 scores) 8–10: borderline case	8	10	7	5
HAD anxiety (0–21 scores) 8–10: borderline case	7	10	6	8
Drug use (%)				
MEDD (mg/day) (med (IQR))	91	**137** *	84	**152** **
Neuromodulators	52	45	50	64
Antidepressants	39	**61** *	32	42
Benzodiazepines	**47** *	55	25	**50** *
Health resources use data (%)				
Emergency department visits	20	36	19	20
Hospitalization	4	5	3	5
Medication changes	48	60	45	45

*Note*: Values are %, mean (SD) or median [IQR]. The highest value is shown in bold.

Abbreviations: HAD: Hospital Anxiety and Depression Scale; MEDD, morphine equivalent daily dose; VAS, Visual Analogue Scale.

* *p* < 0.05 when comparing controls and cases.

** *p* < 0.01 when comparing controls and cases.

^**+**^ *p* < 0.05 when comparing women and men.

**FIGURE 1:**
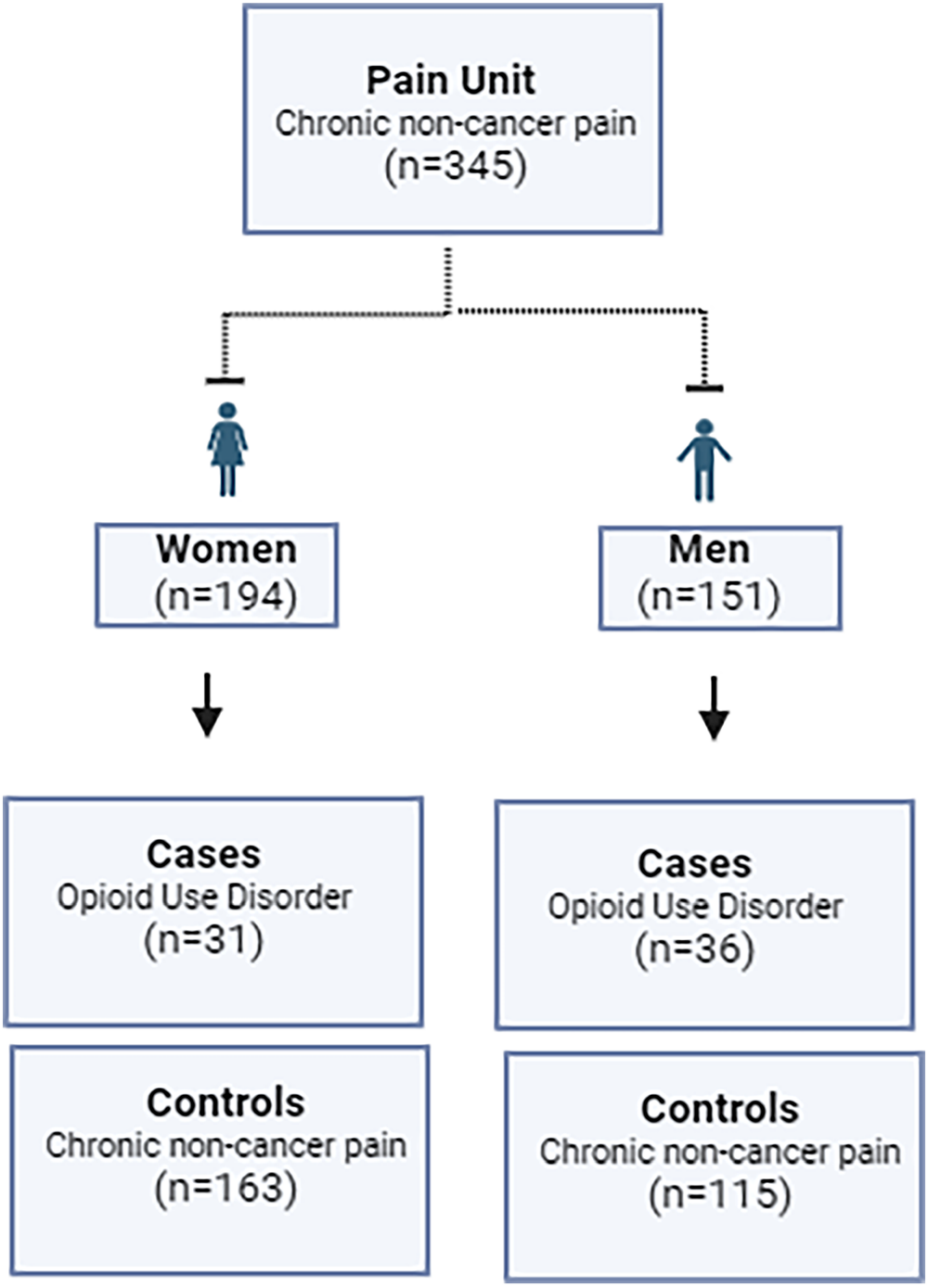
Flow chart of the patients included according to sex and the opioid use disorder as cases or control groups.

Briefly, PCR was performed under standard conditions with biotinylated primers. Pyrosequencing reactions and the DNA methylation quantification of five CpG sites located at their promoter regions (Figure 1) were performed in a PyroMark Q24 System, version 2.0.7 (Qiagen), using appropriate reagents and recommended protocols.

### 2.4 Statistical analysis

Convenience sampling was considered to increase statistical power with a ratio one OUD case to four CNCP controls. This entailed selecting all the available patients from the historic database. The continuous quantitative variables (i.e., pain intensity, pain relief, quality of life and health utility status) in the descriptive analysis are presented as mean ± standard deviation (SD). The discrete variables (i.e., age, HADS scores and AEs) are shown their median and interquartile range (IQR), while categorical data (sex, employment status, anxiety and depression groups, pharmacological prescription) are expressed as percentages. The Kolmogorov–Smirnov test was chosen to perform parametric or non‐parametric tests for comparisons. The comparisons between two given groups of data presenting parametric distributions, such as age, were performed by independent *t*‐test analyses, and an ANOVA test was performed for the analyses by comparing three groups. An analysis of non‐parametric data, such as VAS scores or health utility status, was performed by Mann–Whitney *U* tests for between‐group comparisons. The comparisons for the categorical data were made using the chi‐square goodness‐of‐fit test (*χ* ^2^) and Fisher's exact test. A value of *p* < 0.05 was considered to be statistically significant. Statistical analyses were carried out using the R software package, version 4.03, and the GraphPad Prism software 5.0.

## 3 RESULTS

The number of patients was limited mostly by DNA sample availability. Pre‐screening involved 806 candidates, of whom 460 were limited mainly by DNA sample availability (especially men), unidentifiable or duplicated among databases. Finally, 345 Caucasian patients (OUD cases, *n* = 67; CNCP controls, *n* = 278) were included (Figure 1), as seen in Table S1.

### 3.1 Sex differences in the demographic and clinical data

The 345 subjects (of whom 56% were females) were analysed and mostly Caucasian, middle‐age (64 [51–73] years old) residents in Spain. A summary of sex‐related differences in the demographic and clinical outcomes is presented in Table 1.

In general, age, employment status, quality of life, MEDD and psychotropic prescription were significantly different between cases and controls, with differences also appearing between women and men. Cases were older than controls, with a significant difference of older age for women in both groups. Employment status was significantly different with higher rates of retired status in the controls and work disability in the cases. A lower prevalence of retired status was found in women with OUD with the highest unemployment rate.

The control men showed higher quality of life compared to the cases and independently of sex. However, the women cases exhibited a higher degree of anxiety and depression (mean of 10 scores) with more borderline cases in cases and regardless of sex. In fact, women were prescribed significantly more anxiolytics (controls) and antidepressants (cases) than men.

### 3.2 DNA *OPRM1* methylation differences by sex and opioid use disorder

The DNA methylation values obtained at the five selected CpG sites of the *OPRM1* promoter gene showed dynamic ranges between 6% and 17%. The methylation levels for each site appear in Table S2.

**FIGURE 2:**
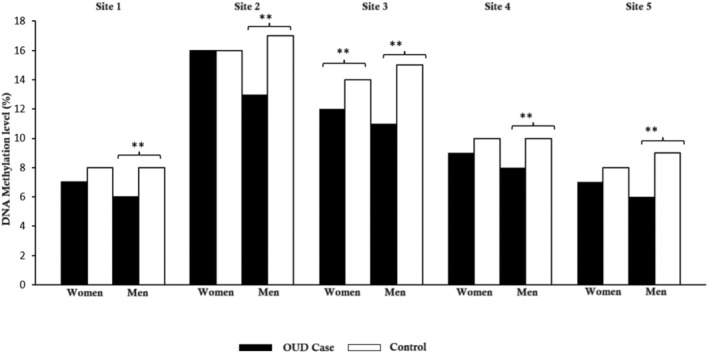
Differences in the DNA methylation levels of the *OPRM1* gene (CpG sites 1–5 located in the promoter region): women versus men and OUD cases versus controls. OUD, opioid use disorder. **p < 0.01.

Significant differences were observed between the OUD case and control groups (see Figure 2). The results revealed that the men with OUD presented significantly lower *OPRM1* DNA methylation levels at all the analysed CpG sites (1–5) compared to the control men. For women, a similar trend was observed and was significant only at the selected CpG‐3. Globally, the men with OUD evidenced significant lower DNA methylation than controls and women.

### 3.3 Association between *OPRM1* DNA methylation levels and clinical outcomes

The regression models analysis showed significant associations between the *OPRM1* DNA methylation values (%) and the different clinical variables. As Table 2 reveals, pain relief was negatively associated with CpG site 1 methylation (*p* = 0.029, *β* = −4.505), while this negative association for AEs like dry skin and loss of appetite was observed with CpG site 4 (*p* = 0.025 *β* = −0.435 and *p* = 0.005 *β* = −0.700, respectively). A positive correlation was found between depression (HAD or patient‐reported scores), dry mouth, oedema and the total AEs. No association was detected with CpG sites 3 and 5.

**TABLE 2:** Associations between *OPRM1* DNA methylation (CpG sites 1–5) and clinical outcomes in terms of effectiveness and tolerability as adverse events (AEs) due to the regression model results.

CpG	Site 1		Site 2		Site 3		Site 4		Site 5	
	*p*	Est	*p*	Est	*p*	Est	*p*	Est	*p*	Est
Pain relief	**0.029**	−4.505	0.140	1.435	0.361	1.417	0.314	−1.971	0.358	1.665
Depression	0.775	−0.102	**0.043**	0.453	0.187	0.425	0.077	−0.738	0.613	−0.177
Total AEs	0.570	0.128	**0.018**	0.269	0.211	−0.221	0.140	−0.337	0.738	0.070

*Note*: Bold emphasis indicates significant differences.

Abbreviations: Est, estimate; *p*, *p*‐value.

## 4 DISCUSSION

The present study observed sex‐related differences in OUD that were consistent with previous studies.^27^, ^28^ The men with OUD evidenced significantly lower *OPRM1* DNA methylation than the controls and women. This low *OPRM1* DNA methylation correlated with less pain relief, depression and a different pattern of AEs in cases. Moreover, the cases obtained different psychosocial outcomes (age, employment status), quality of life and drug use (higher MEDD and psychotropic drug prescription) than the controls. These findings highlight the complex interplay among pain, dependence, the environment^29^ and sex^8^, ^27^, ^30^ and indicates the possibility of reducing or preventing the negative consequences of chronic pain and OUD. We insist that a successful OUD crisis response should highlight the importance of understanding sex differences in opioids use patterns.

It is well‐known that the environment (e.g., diet, exposure to chemicals or toxins, psychological stress, etc.) can influence the epigenome and gene expression.^31^ Indeed *OPRM1* as the main receptor of opioids plays an important role in the pharmacological process of opioids in rodents and humans.^32^, ^33^, ^34^ It has been demonstrated that sex differences in the effects of peripheral *OPRM1* agonists are partly mediated by sex differences in changes in *OPRM1* expressions^35^ and by tolerance/dependence results from different adaption strategies (chronic systemic morphine influences *OPRM1* splice variant mRNA levels) in men and women.^36^ Using immunohistochemistry, it was observed that male rats had a significantly higher expression of MOR compared with cycling females. Here, a significant reduction in the effects of systemic morphine was evidenced, in males only, after a selective lesion of MOR‐expressing neurons.^37^ These results provide a mechanism for sex differences in morphine potency. Even more, inflammation and cytokines induced up‐regulation of MOR in trigeminal ganglia in a sex‐dependent manner modulated by testosterone.^38^ However, the identification of specific factors associated with either the individual opioid response or side effect vulnerability has only been initiated.^39^ The molecular mechanisms that explain why some individuals develop negative consequences associated with prolonged prescription opioid use, including dependence, cognitive or sleep problems, are still poorly understood.

Furthermore, recent data from our region showed that younger patients, those with a lower employment status, those taking high opioid doses and on psychotropic co‐prescription were more vulnerable to OUD.^40^ Thus, prevention and treatment programs should be tailored to consider sex and age differences in sources of opioids^41^ as obtained from physicians, drug dealers or friends/relatives. Incident opioid overdoses have also been related to lower socio‐economic status (due to completed level of education and having benefited from social welfare) in a retrospective study based on Swedish national register data.^42^ An US survey (*n* = 1229) reported that 80% of CNCP patients on ≥50 mg MEDD continued higher dose opioid use for 1 year, regardless of reported problems, concerns, side effects, pain reduction or perceived helpfulness.^43^ These results suggest the difficulty of OUD tapering procedures and the need for better understanding as key factors for supporting preventive strategies.

Although our results explain the relation between sex‐related differences in OUD from the *OPRM1* methylation perspective, there were still a few limitations. First, the retrospective design limited the data collection of some variables. Additionally, opioids imply increased susceptibility to abuse, particularly when too many opioid drugs are prescribed for conditions that are not assumed to be treated by opioids or are not monitored enough.^1^ Second, other unmeasured outcomes (body mass index, hormones, lifestyle) could have contributed to the observed differences. Third, our sample size was limited to the DNA available in a single hospital. This could bias the external validity of this study by being more relevant than pain itself. Fourth, the *COMT* promoter site obtained methylation values close to zero with very little variability. Therefore, the methylation patterns of other, but still unexplored genes seem more relevant. Finally, OUD diagnoses were made according to the *Diagnostic and Statistical Manual of Mental Disorders* (5th edition; DSM‐V), through almost 2 of the 11 behavioural or psychological criteria were positive during a 12‐month period with a persistent desire or unsuccessful efforts to cut down or control opioid use.^44^ However, an analysis was done independently of the scores and causes of OUD diagnoses. Improved translational relevance will also require focusing more on genetic/epigenetic impacts, together with interventions that address co‐occurring mental health conditions and psychosocial stress for those patients with OUD. Such work will significantly advance our understanding of how opioids cause persistent changes to brain function and will provide a platform on which to develop interventions for preventive or for treating OUD strategies.

Our data showed an association between *OPRM1* gene methylation values in the men with OUD with an impact on other clinical and safety outcomes. For both sexes, some psychosocial factors like opioid and psychotropic use were higher in the OUD cases than in the controls and were significant in men. Our results highlight the importance of understanding sex and gender differences in the current opioid epidemic. This could allow healthcare practitioners to take prevention measures when chronic opioid exposure is needed in CNCP patients and to design treatment programs that should be tailored to each patient.

## AUTHOR CONTRIBUTIONS

*Conceptualization*: Ana M. Peiró and Javier Muriel. *Methodology*: Laura Agulló, Mónica Escorial and Javier Muriel. *Formal analysis*: Laura Agulló, Juan Sandoval and Samantha Orutño. *Investigation*: Laura Agulló, Mónica Escorial and Ana M. Peiró. *Resources*: Ana M. Peiró. *Curation*: Laura Agulló, César Margarit and Javier Muriel. *Writing—original draft preparation*: Laura Agulló and Ana M. Peiró. *Writing—review and editing*: all authors. *Visualization*: Laura Agulló and Ana M. Peiró. *Supervision*: Ana M. Peiró. *Project administration*: Laura Agulló, Ana M. Peiró and Javier Muriel. *Funding acquisition*: Ana M. Peiró. All authors have read and agreed to the published version of the manuscript.

## CONFLICT OF INTEREST STATEMENT

The authors declare no conflicts of interest.

## ETHICS STATEMENT

This study was approved by the Ethics Committee of the Dr. Balmis Alicante University Hospital.

## Supporting information


**Table S1.** The primers used in the pyrosequencing assay.

**Table S2.** Analysis of the genetic variants and DNA methylation (CpG site 1–5) of the *OPRM1* gene by sex when comparing the case group to Opioid Use Disorder (OUD) and the controls.

## Data Availability

The data that support the findings of this study are available on request from the corresponding author, AMP. The data are not publicly available due to their containing information could compromise the privacy of research participants.
