# Correction to “Epigenetic and Sex Differences in Opioid Use Disorder in Chronic Pain: A Real‐World Study Linked With OPRM1 DNA Methylation”

**DOI:** 10.1111/adb.70030

**Published:** 2025-03-17

Agulló, L. , Escorial, M. , Orutño, S. , Muriel, J. , Sandoval, J. , Margarit, C. , & Peiró, A. M. (2024). Epigenetic and Sex Differences in Opioid Use Disorder in Chronic Pain: A Real‐World Study Linked With OPRM1 DNA Methylation. Addiction Biology, 29(7), e13422.38949208 10.1111/adb.13422PMC11215788

The correct name of one of the authors is Samanta Ortuño‐Miquel, not Samantha Orutño as it is currently presented.

We apologize for this error.

