# Supplementary material for: Epigenetic and sex differences in opioid use disorder in chronic pain: A real‐world study linked with OPRM1 DNA methylation
Source: Addict Biol. 2024 Jul 1;29(7):e13422. doi: 10.1111/adb.13422 (PMC11215788; doi:10.1111/adb.13422)
Supplement: Supplementary file 1 — Table S1. The primers used in the pyrosequencing assay. [file ADB-29-e13422-s002.docx]

| **Table S1.** The primers used in the pyrosequencing assay. | | |
| --- | --- | --- |
| **Primer** | **Sequence** | **CpG sites** |
| *OPRM1*_F1 | 5´-GGATTGGTTTTTGTAAGAAATAGTAGG-3´ |  |
| *OPRM1*_R1 | 5´-ATACRCCAAAACATCAATACAATTACTAAC-3´ |  |
| ***OPRM1*_S1** | **5´-AAGTTTYGGTGTTTTTGGTTA-3´** | **CpG 7 – 11** |
