# Supplementary material for: Epigenetic and sex differences in opioid use disorder in chronic pain: A real‐world study linked with OPRM1 DNA methylation
Source: Addict Biol. 2024 Jul 1;29(7):e13422. doi: 10.1111/adb.13422 (PMC11215788; doi:10.1111/adb.13422)
Supplement: Supplementary file 2 — Table S2. Analysis of the genetic variants and DNA methylation (CpG site 1–5) of the OPRM1 gene by sex when comparing the case group to Opioid Use Disorder (OUD) and the controls. [file ADB-29-e13422-s001.docx]

| **Table S2.** Analysis of the genetic variants and DNA methylation (CpG site 1-5) of the *OPRM1* gene by sex when comparing the case group to Opioid Use Disorder (OUD) and the controls. | | | | |
| --- | --- | --- | --- | --- |
|  | **Women** | | **Men** | |
|  | **Controls**  163 (84%) | **OUD Cases**  31 (16%) | **Controls**  115 (76%) | **OUD Cases**  36 (24%) |
| *OPRM1 Genotype (%)* | | | | |
| AA | 62 | 68 | 65 | 67 |
| AG | 34 | 29 | 31 | 33 |
| GG | 4 | 3 | 4 | 0 |
| *OPRM1 DNA Methylation (%)* | | | | |
| Site 1 | 8 | 7 | 8 | **6**** |
| Site 2 | 16 | 16 | 17 | **13**** |
| Site 3 | 14 | **12**** | 15 | **11**** |
| Site 4 | 10 | 9 | 10 | **8**** |
| Site 5 | 8 | 7 | 9 | **6**** |
| ** Denotes p<0.05 and ** depicts p<0.01 when comparing the controls and OUD cases.* | | | | |
